# Muscle oxygenation and time to task failure of submaximal holding and pulling isometric muscle actions and influence of intermittent voluntary muscle twitches

**DOI:** 10.1186/s13102-022-00447-9

**Published:** 2022-03-30

**Authors:** Silas Dech, Frank N. Bittmann, Laura V. Schaefer

**Affiliations:** Department of Sport and Health Sciences, Regulative Physiology and Prevention, Human Science Faculty, University of Potsdam, Karl-Liebknecht-Str. 24-25, 14479 Potsdam, Germany

**Keywords:** Oxygen saturation, Microvascular blood filling, Isometric contraction, Isometric muscle action, Holding isometric muscle action, Pulling isometric muscle action, Pushing isometric muscle action, Time to task failure, Muscle twitch

## Abstract

**Background:**

Isometric muscle actions can be performed either by initiating the action, e.g., pulling on an immovable resistance (PIMA), or by reacting to an external load, e.g., holding a weight (HIMA). In the present study, it was mainly examined if these modalities could be differentiated by oxygenation variables as well as by time to task failure (TTF). Furthermore, it was analyzed if variables are changed by intermittent voluntary muscle twitches during weight holding (Twitch). It was assumed that twitches during a weight holding task change the character of the isometric muscle action from reacting (≙ HIMA) to acting (≙ PIMA).

**Methods:**

Twelve subjects (two drop outs) randomly performed two tasks (HIMA vs. PIMA or HIMA vs. Twitch, n = 5 each) with the elbow flexors at 60% of maximal torque maintained until muscle failure with each arm. Local capillary venous oxygen saturation (SvO_2_) and relative hemoglobin amount (rHb) were measured by light spectrometry.

**Results:**

Within subjects, no significant differences were found between tasks regarding the behavior of SvO_2_ and rHb, the slope and extent of deoxygenation (max. SvO_2_ decrease), SvO_2_ level at global rHb minimum, and time to SvO_2_ steady states. The TTF was significantly longer during Twitch and PIMA (incl. Twitch) compared to HIMA (*p* = 0.043 and 0.047, respectively). There was no substantial correlation between TTF and maximal deoxygenation independently of the task (r = − 0.13).

**Conclusions:**

HIMA and PIMA seem to have a similar microvascular oxygen and blood supply. The supply might be sufficient, which is expressed by homeostatic steady states of SvO_2_ in all trials and increases in rHb in most of the trials. Intermittent voluntary muscle twitches might not serve as a further support but extend the TTF. A changed neuromuscular control is discussed as possible explanation.

**Supplementary Information:**

The online version contains supplementary material available at 10.1186/s13102-022-00447-9.

## Background

Isometric muscle actions can be performed during two different tasks. On the one hand, a person can apply force by pushing against or pulling on a stable resistance (e.g., common determination of the maximal voluntary isometric contraction (MVIC)). The person acts with the attempt to shorten the muscle but this is prevented by the stable resistance. Hence, the muscle action stays isometric [1]. This refers to a pushing or pulling isometric muscle action (PIMA) [2]. It has also been called “force task” [3–5] “restrained task” [6], or “concentrically loaded isometric contraction” [1]. On the other hand, a person can react to an external load by holding a position. The person reacts with the attempt to resist the externally provoked lengthening [1] by adapting to the applied load. The external load can be, e.g., a constant weight or increasing force for assessment of the maximal isometric Adaptive Force [7, 8]. Such muscle action refers to a “holding isometric muscle action” (HIMA) [2]. It was also termed “position task” [3–5], “postural task” [6], or “eccentrically loaded isometric contraction” [1].

In previous studies, both isometric tasks could partly be differentiated by various variables [2–5, 7, 9–11]. Most consistently, the time to task failure (TTF) was found to be significantly shorter during HIMA compared to PIMA at low intensities (15%, 20% and 30% of the MVIC) performed by arm flexor muscles in a horizontal forearm position [3, 4, 9, 11]. With a vertically positioned forearm and at intensities of 45%, 60% and 80% conflicting results have been found [2–4, 10]. Other variables which had shown differences between isometric tasks were amplitudes of electromyography (EMG) or mechanomyography (MMG) at exhaustion (PIMA > HIMA) [2, 3], normalized EMG power (PIMA > HIMA) [4] as well as MTG power (HIMA > PIMA) [2] in special frequency bands, mean arterial blood pressure (HIMA > PIMA) [3], glucose uptake in young men (HIMA > PIMA) [5] and the maximal torque (PIMA > HIMA) [7].

However, some studies did not reveal a difference between HIMA and PIMA in any of their analyzed parameters: MVIC, TTF and mean heart rate relative to rest for the biceps brachii muscle [12]; motor unit discharge characteristics and extent of motor unit synchronization at 4.4% of the MVIC (PIMA) or 3.8% of the one-repetition maximum (HIMA) for the first dorsal interosseus muscle [6] as well as EMG amplitudes at 20%, 30%, 40%, and 50% of the MVIC for the soleus muscle [1].

Regarding muscle oxygenation, Booghs et al. (2012) analyzed tissue oxygenation index (TOI) and normalized total hemoglobin index (nTHI) of the biceps brachii muscle between HIMA and PIMA at 20% and 60% of the MVIC [11]. They found similar behaviors for both tasks at both intensities. However, at 60%, the TOI seemed to decrease more during PIMA compared to HIMA. A statistical comparison was not provided.

The main objective of the present pilot study was to compare the muscle oxygenation and blood filling of microvessels between HIMA and PIMA maintained until muscle failure (fatiguing task). Previous studies have revealed that during isometric muscle actions homeostatic steady states in oxygen saturation are possible even at submaximal and maximal intensities [11, 13–17]. Thus, not only the extent and slopes of deoxygenation but also the time to leveling off into a steady state was compared between HIMA and PIMA in the present study. Our research group has differentiated two behavioral types in the regulation of local capillary venous oxygen saturation (SvO_2_) and relative hemoglobin amount (rHb) in superficial muscle tissue [13]. In type I, SvO_2_ and rHb generally decrease and level off into steady states. Both parameters behave nearly parallel to each other. In contrast, an increase in rHb despite a further decrease in SvO_2_ is the main characteristic of type II. Thus, the behavior is partly inverse. The occurrence of type I or type II was also documented in the present study. Our research group suggested that the type depends on the oxygenation level [17]. The increase in rHb after reaching its global minimum (type II) might be triggered if SvO_2_ level drops below a threshold around 59% [17]. Thus, the oxygenation level at the moment of global rHb minimum was compared between HIMA and PIMA here.

Furthermore, TTF was compared between HIMA and PIMA since it seems to be one of the most promising variables as mentioned above. The analyses should gain further data concerning the supposed distinction of two different isometric muscle actions [1–6, 12].

It must be noted that a holding task per se does not automatically prevent a muscle shortening (e.g., overcoming an applied weight). If tolerated, minimal concentric contractions are possible to compensate for a prior lengthening. At macro level this still might be interpreted as an isometric muscle action. In this regard, intermittent voluntary muscle twitches (Twitch) were performed by a another group of subjects during a weight holding task in the present study. Subjects were instructed to twitch rapidly and shortly. Caused by that kind of minor concentric contractions, the character of isometric muscle action might be changed also during the isometric phases [2]: from reacting (HIMA) to acting (PIMA). It was also questioned, if the muscle twitches have an influence on all above-mentioned variables. From a general understanding, isometric muscle actions performed at submaximal intensities were supposed to lead to a restriction of oxygen and blood supply caused by high intramuscular pressures [18–22]. Rapid but short auxotonic contractions with minimal motion of the limb (Twitch) change muscle length and tension temporarily. This might support the capillary blood flow by serving as a kind of pump. Consequently, blood and oxygen supply could be changed. The TTF might also be different because of an altered neuromuscular control.

## Materials and methods

### Subjects

Both biceps brachii muscles of twelve subjects were examined in this pilot study. Two of these had to be excluded due to pain or discomfort during the trials (*n* = 10; 8 males, 2 females, mean age ± standard deviation (SD) = 30.70 ± 11.67 years, 72.70 ± 11.00 kg, 1.78 ± 0.08 m, BMI: 22.84 ± 2.00 $\frac{\mathrm{kg}}{{\mathrm{m}}^{2}},$ two lefties).

### Measuring technique

The valid and reliable O2C spectrophotometer (Oxygen To See; LEA© Medizintechnik GmbH, Gießen, Germany) mainly recorded the local capillary venous oxygen saturation (SvO_2_, not to be confused with the systemic mixed venous oxygen saturation) and the relative hemoglobin amount (rHb) [23–27]. The device sent light (650–810 nm, 1 nm resolution) into the superficial muscle tissue through the measuring probe (“LF-3”, source-detector separation: 14.5 mm, tissue penetration depth: ~ 12 mm). The probe was fixed above the biceps brachii muscle belly along its fibers. The amount of backscattered light and changed wavelength was used for calculating rHb in arbitrary units (AU) in dependence of the absorption rate. SvO_2_ (in %, absolute measurement) was calculated as a ratio of primarily oxygenated and deoxygenated hemoglobin as well as myoglobin. The sampling rate was 40 Hz. To minimize light effects on the probe, the room light was dimmed. Even though the O2C device mainly analyze the capillary venous system, the influence of arterial blood cannot be excluded completely [28–31]. Furthermore, myoglobin also influences measurements during exercise although in a lower extent compared to rest [32, 33].

### Study design and overall procedure

All subjects started with a MVIC-test with one arm randomly selected (coin toss). Two different determination methods of the MVIC were utilized according to the subsequent fatiguing tasks. The first group performed HIMA and PIMA (HP-group), the second group performed HIMA and Twitch (HT-group) (specific description see below). The authors find this acceptable since comparisons were only made within subjects. The sequence of fatiguing tasks was randomized by coin toss. The rest between fatiguing tasks was at least 3 min. Afterwards, the same procedure was applied to the other arm. An intensity of 60% of the MVIC was chosen for the fatiguing tasks according to Booghs et al. (2012) [11], and because it might lead to high intramuscular pressures to restrict the blood and oxygen supply [18]. The recording of parameters (SvO_2_ and rHb) started 10 s before and lasted until two minutes after every task.

### Procedures of the HP-group trials

**Fig. 1:**
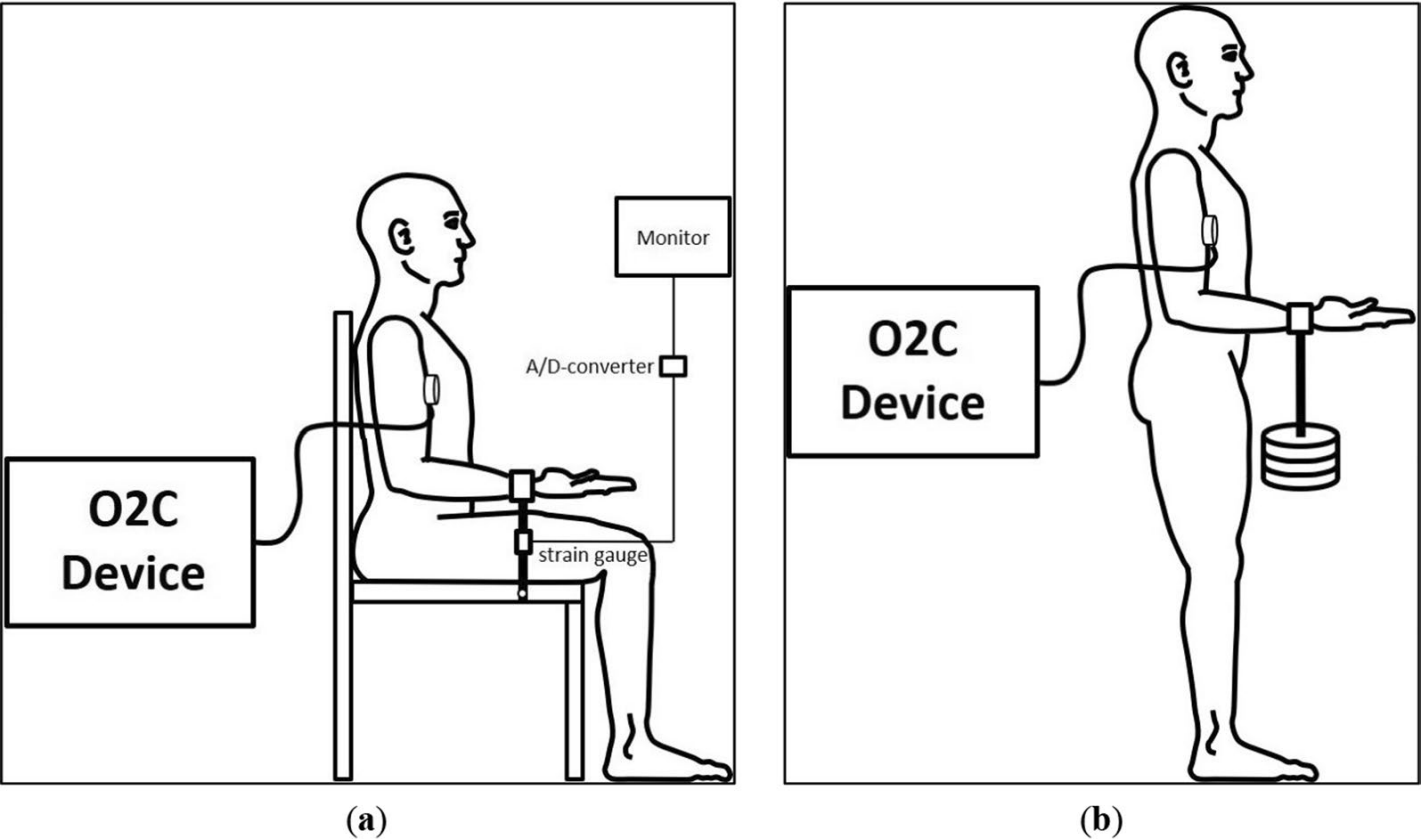
Positioning and set up during the tasks, **a** pulling isometric muscle action (PIMA), **b** holding isometric muscle action (HIMA) and Twitch, reprinted from Dech et al. (2021), with permission [17]

During the MVIC-tests, the subjects of the HP-group sat on a measuring chair in an upright position. They pulled two times as strong as possible on a cuff which was connected to a fixed strain gauge (Fig. 1a). The rest between trials was at least two minutes. The cuff was applied 2–3 cm proximal to the wrist crease of the subject whose upper arm was in contact with the thorax (anteversion–retroversion 0°, adduction–abduction 0°). The elbow joint was flexed (90°) and the forearm was maximally supinated. The highest measured force value of the two trials was determined as MVIC.

During fatiguing PIMA (Fig. 1a), the arm and sitting positions were identical to the MVIC-tests. The subjects pulled on a fixed strain gauge and maintained the target force of 60% of the MVIC for as long as possible. For that, they had visual feedback (pointer) on a monitor. As soon as the force remained below the target for 2 s, the rater prompted the subject to stop the task.

During fatiguing HIMA, the subjects had to hold the respective weight for as long as possible with the same arm position but while standing, which allows the weight to hang free (Fig. 1b). The weight was taken off as soon as the elbow angle exceeded 90° for 2 s, assessed by the rater subjectively.

### Procedures of the HT-group trials

The MVIC-test of the HT-group was performed by holding a weight while standing and using the same arm and cuff position as described in the HP-group section (Fig. 1b). The weights were added progressively within maximal five steps (accuracy: ± 1 kg). The test started with an estimated appropriate first weight. The rater hooked the respective weight onto the cuff. The rest between steps had to be sufficient for the subject (30 s–2 min., depending on the load). The highest weight which could be held for 1 s was determined as MVIC. This procedure did not achieve the same accuracy as determined by strain gauge measurements but was chosen in favor of the fatiguing weight holding tasks.

The fatiguing HIMA was performed identically as described for the HP-group. The same applies for the fatiguing Twitch task except for additional intermittent voluntary contractions (muscle twitches) every 7 s. For that, an acoustic signal was given. The subjects were instructed to perform twitches rapidly but with a minimal excursion of the forearm which was visually inspected by the rater. An objective determination of the twitches was not performed (see limitations). The rater took off the weight as soon as the subject could not twitch again or if the elbow angle exceeded 90° for 2 s, visually assessed by the rater.

### Data processing

All SvO_2_ and rHb curves were smoothed by using the software in NI DIAdem™ 2017 (moving average, maximal smoothing width: 50). The following variables were extracted for each trial:

- Variable (1.): SvO_2_ baseline value (in %) was quantified by the arithmetic mean (M) of the values of the first 10 s when the arm was held in measurement position (described above).
- Variable (2.): Maximal deoxygenation (max. SvO_2_ decrease) was determined as the difference between the baseline and the minimum of SvO_2_. Values are presented in percent points (pp) and additionally in % related to the respective baseline.
- Variable (3.): Slope of initial linear SvO_2_ decrease after start of loading (SvO_2_ slope) was quantified by the slope of the least square regression line. According to Felici et al. (2009) [34], interval limits for calculating the slope were set respect of the start and end point of the first long negative plateau of the first derivative of the smoothed SvO_2_ curve.
- Variable (4.): SvO_2_ level at global minimum of rHb (SvO_2_ at rHb min.) corresponds to SvO_2_ value at the reversal point (RP) in type II.
- Variable (5.): Time to leveling off into a steady state of SvO_2_ (TSS) is the time period in s from start of loading to the end of the initial linear phase (start of leveling off into a steady state).
- Variable (6.): Time to task failure (TTF) was defined as the time period in s from start to end of loading.

**Fig. 2:**
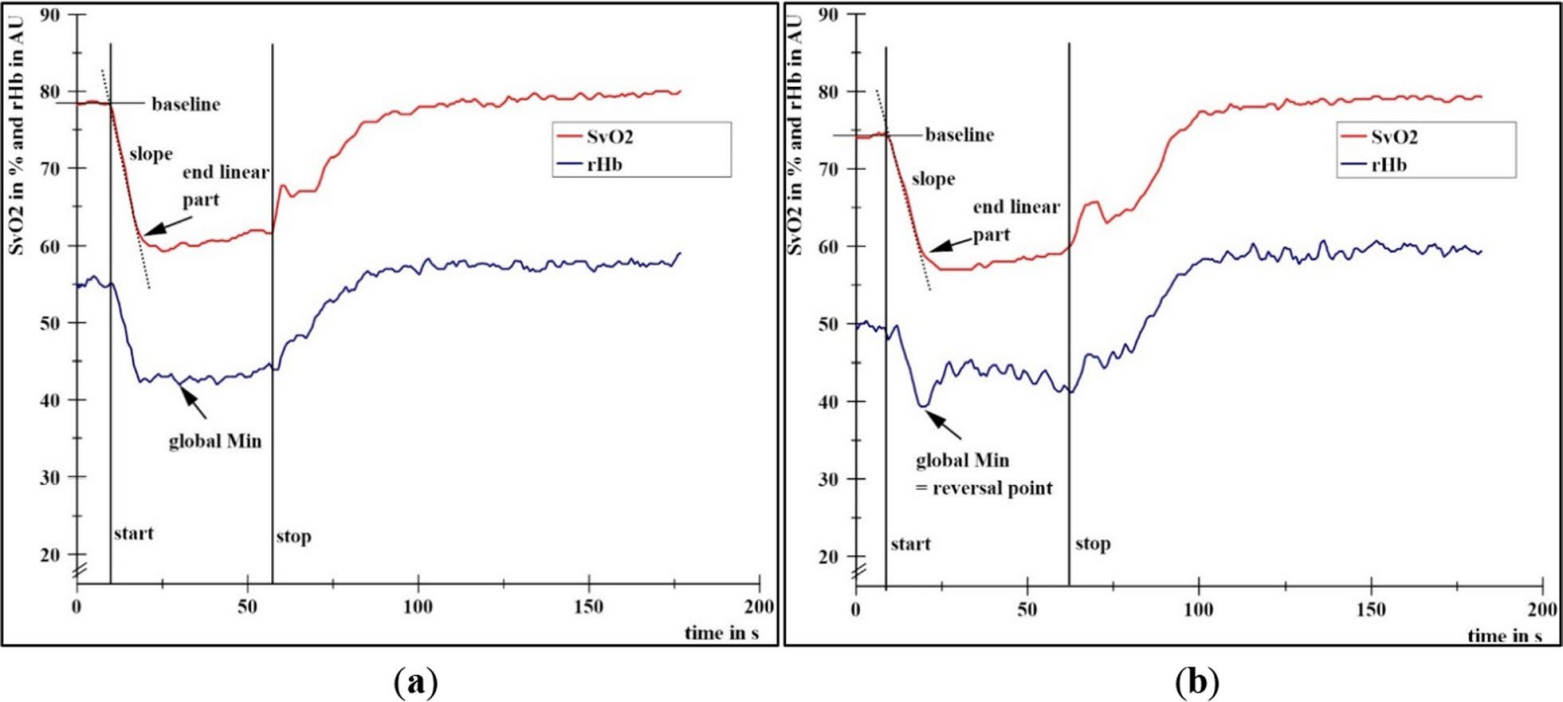
Curve examples of the local capillary venous oxygen saturation of hemoglobin (SvO_2;_ red) and the relative hemoglobin amount (rHb, blue) during two fatiguing isometric muscle actions at 60% of the MVIC of the right biceps brachii muscle of one female (37 years, 1.60 m, 58 kg): **a** HIMA (type I) and **b** PIMA (type II). Start and stop of loading are indicated by vertical lines. Baseline, slope, end of linear slope part and the global minimum are given. The global Min in **b** refers to the reversal point. All curves were smoothed (moving average, maximal smoothing width: 50)

According to Dech et al. (2020) [13], the curves were also assigned visually to type I (parallel behavior of SvO_2_ and rHb, Fig. 2a) or type II (increase in rHb after the RP, Fig. 2b). The assignment and all mentioned variables (1.–6.) of each trial can be found in Additional file 1: Table S1.

### Statistical analyses

IBM SPSS Statistics 26 was used for statistical analyses. Due to discomfort triggered by the cuff, the trials of two subjects and the trials of one side of two other subjects had to be excluded. Another trial (Twitch) was excluded because the participant failed to perform the twitches rapidly and shortly. In total, 36 trials of ten subjects were included for statistical analyses (HP and HT n = 9 trials for each task).

All mentioned variables (1.–6.) were compared between fatiguing tasks but only within subjects (HIMA vs. PIMA and HIMA vs. Twitch as well as HIMA vs. PIMA incl. Twitch). Both arms were considered together in all analyses, because MVICs of right (70.02 ± 23.83 Nm) and left arm (69.31 ± 21.69 Nm) did not differ significantly (t(11) = 0.80, p = 0.442).

All data were normally distributed (Shapiro–Wilk-test, p > 0.05), except for baseline values of Twitch (p = 0.017) and max. SvO_2_ decrease of all HIMAs (p = 0.019). Regarding normal distributed variables, analyses of differences were made by parametric tests (t-tests for dependent samples). Comparisons including not normally distributed variables were made by exact Wilcoxon signed-rank test. Numbers of type I and type II behaviors per task were compared by Chi-squared test. An alpha error of 5% was chosen for all tests.

Pearson’s correlation coefficients (r) were calculated between TTF (6.), max. SvO_2_ decrease (2.) and SvO_2_ slope (3.) of all 36 trials.

## Results

Tasks did not differ significantly regarding oxygenation variables (1.–5.). In regard to TTF (6.), HIMA and PIMA did not differ significantly despite a trend in favor of PIMA. The TTF during Twitch was about 10 s longer than during HIMA (*t*(8) = − 2.40, *p* = 0.043). The comparison of all HIMAs and PIMAs (incl. Twitches) also showed a significant difference (*t*(17) = − 2.15, *p* = 0.047). The inference statistics of all described variables can be found in Table 1.

**Table 1:** Inference statistics of within-subject comparisons between tasks

Variable	Group	Fatiguing task	Mean ± SD	t-values (df)/z-values	Significance level
(1.) Baseline value in %	HP	HIMA	73.78 ± 6.63	*t*(8) = − 1.53	*p* = 0.164
		PIMA	77.44 ± 4.06		
	HT	HIMA	74.61 ± 6.98	*z* = − 0.30, *n* = 9	*p*_*exact*_ = 0.820
		Twitch	74.00 ± 5.01		
	Both	HIMA	74.19 ± 6.62	*z* = − 1.15, *n* = 18	*p*_*exact*_ = 0.265
		PIMA & Twitch	75.72 ± 4.77		
(2.) max. SvO_2_ decrease in pp (% to baseline)	HP	HIMA	16.02 ± 7.75 (21.71 ± 10.50)	*t*(8) = − 1.73	*p* = 0.121
		PIMA	20.61 ± 10.16 (26.61 ± 13.12)		
	HT	HIMA	30.33 ± 14.60 (40.65 ± 19.57)	*t*(8) = − 0.12	*p* = 0.909
		Twitch	30.65 ± 16.76 (41.42 ± 22.65)		
	Both	HIMA	23.17 ± 13.52 (31.23 ± 18.22)	*z* = − 0.81, *n* = 18	*p*_*exact*_ = 0.442
		PIMA & Twitch	25.63 ± 14.40 (33.85 ± 19.02)		
(3.) SvO_2_ slope in pp s^−1^	HP	HIMA	− 2.21 ± 1.51	*t*(8) = 0.55	*p* = 0.598
		PIMA	− 2.41 ± 1.59		
	HT	HIMA	− 3.63 ± 1.56	*t*(8) = − 0.94	*p* = 0.373
		Twitch	− 3.11 ± 1.31		
	Both	HIMA	− 2.79 ± 1.60	*t*(17) = − 0.11	*p* = 0.913
		PIMA & Twitch	− 2.76 ± 1.46		
(4.) SvO_2_ at rHb min. in %	HP	HIMA	61.96 ± 3.87	*t*(8) = − 0.90	*p* = 0.392
		PIMA	62.42 ± 4.56		
	HT	HIMA	58.66 ± 2.61	*t*(8) = − 0.31	*p* = 0.768
		Twitch	58.81 ± 3.04		
	Both	HIMA	60.31 ± 3.63	*t*(17) = − 0.89	*p* = 0.388
		PIMA & Twitch	60.62 ± 4.20		
(5.) TSS in s	HP	HIMA	7.72 ± 3.01	*t*(8) = − 0.5	*p* = 0.630
		PIMA	8.45 ± 2.72		
	HT	HIMA	7.61 ± 2.61	*t*(8) = − 1.01	*p* = 0.344
		Twitch	8.78 ± 2.92		
	Both	HIMA	7.67 ± 2.74	*t*(17) = − 1.05	*p* = 0.309
		PIMA & Twitch	8.62 ± 2.74		
(6.) TTF in s	HP	HIMA	44.80 ± 18.06	*t*(8) = − 0.90	*p* = 0.394
		PIMA	50.33 ± 9.46		
	HT	HIMA	42.63 ± 7.64	*t*(8) = − 2.40	***p* = 0.043**
		Twitch	52.78 ± 11.61		
	Both	HIMA	43.72 ± 13.50	*t*(17) = − 2.15	***p* = 0.047**
		PIMA & Twitch	51.55 ± 10.35		

HIMA, holding isometric muscle action; max. SvO_2_ decrease, maximal deoxygenation; PIMA, pulling isometric muscle action; rHb, relative hemoglobin amount; SvO_2_, local capillary oxygen saturation; SvO_2_ at rHb min., SvO_2_ level at global minimum of rHb; SvO_2_ slope, slope of initial linear SvO_2_ decrease; TTF, time to task failure; TSS, time to leveling off into a steady state of SvO_2_

Significant differences are in bold

Figure 2 illustrates the two occurred behavioral patterns (type I and II) as described in detail by Dech et al. [13].

Based on curve shapes, Table 2 shows the categorization of all fatiguing tasks and MVIC-tests into type I (n = 9) and type II (*n* = 27). The amounts of trials assigned to type I and type II were similar between HIMA and PIMA (*χ*^*2*^ (1, *n* = 18) = 0.23, *p*_*exact*_ = 1.00 as well as HIMA and Twitch (χ^2^ (1, *n* = 18) = 0.00, *p*_*exact*_ = 1.00). The assignment was also similar overall trials (HIMA vs. PIMA (incl. Twitch): χ^2^ (1, *n* = 36) = 0.15, *p*_*exact*_ = 1.00.

**Table 2:** Number (n) of type I and type II behaviors of the local capillary venous oxygen saturation and blood filling during the tasks separated by groups and overall trials of ten subjects

Task	HP-group		HT-group		Overall trials	
	HIMA	PIMA	HIMA	Twitch	HIMA	PIMA (incl. Twitch)
Type I (n = 9)	4	3	1	1	5	4
Type II (n = 27)	5	6	8	8	13	14
n = 36	9	9	9	9	18	18

HIMA, holding isometric muscle action; PIMA, pulling isometric muscle action

Regarding all 36 trials, correlation coefficients were *r* = 0.31 (*p* = 0.069) between TTF and SvO_2_ slope; *r* = − 0.13 (*p* = 0.463) between TTF and max. SvO_2_ decrease and *r* = − 0.88 (*p* < 0.001) between SvO_2_ slope and max. SvO_2_ decrease.

## Discussion

### Muscle oxygenation during different muscle actions

In respect of a possible objective distinction of two isometric modalities, oxygenation variables were compared between HIMA and PIMA. No significant differences were found regarding the maximal deoxygenation, saturation slopes, saturation level at global minimum of rHb and time to leveling off into a saturation steady state. This indicates HIMA and PIMA cannot be discriminated by these variables on the basis of the small sample size investigated here. Regarding the saturation slopes, the result is in accordance with Booghs et al. [11]. They utilized the near infrared spectroscopy technique (NIRS), which is comparable with our used light spectrometry [13, 17]. To our best knowledge, the remaining oxygenation variables were considered for the first time regarding the comparison of HIMA and PIMA.

As generally accepted, isometric muscle actions should restrict the capillary blood flow due to the high intramuscular pressure already at low intensities [19, 20]. However, the occlusion threshold might vary between individuals and muscles [35]. In contrast, auxotonic contractions possibly support oxygen and blood supply due to the reduced muscle tension during the lowering phase. Comparisons between HIMA and Twitch did not show any significant differences in the analyzed variables of the present study. Thus, the curve characteristics of SvO_2_ and rHb seem not to be influenced by twitches. Otherwise, the minimal motion of the limb caused by rapid voluntary muscle contractions might not be enough to change the muscle oxygenation and blood filling of microvessels significantly.

Previous studies revealed that increases in capillary blood filling (rHb) [13, 15, 34, 36–38] and homeostatic steady states in the oxygen saturation [11, 13–16] are possible already during isometric muscle actions without twitches. These data suggest a probable maintenance of capillary blood flow. Recently, it was discussed that this could be achieved by the anatomical placement of capillaries within muscle tissue and the oscillatory behavior of muscle fibers during isometric muscle actions [17].

The behavior of oxygen saturation and blood filling can be differentiated by two types [13]; indicated by the SvO_2_ level at global minimum of rHb [17]. We have suggested a threshold of around 59% whereby values above this threshold are associated with type I (parallel behavior of SvO_2_ and rHb) [17]. In contrast, if the saturation decreases below that threshold, rHb starts to increase which is related to type II. Such behavior might reflect a protective measure to impede a further deoxygenation as discussed previously [17]. The distribution of type I and type II assigned measurements reflects a qualitative behavior of the measured parameters SvO_2_ and rHb. In the presented study, extents of deoxygenation and SvO_2_ levels at global minimum of rHb did not differ significantly between HIMA and PIMA as well as not between HIMA and Twitch. The amount of type I and type II assigned measurements are, as a consequence, not significantly different (Table 2). Thus, the occurrence of type I and type II seems to be independent of the isometric task.

At last, a high and significant negative correlation (r = − 0.88) between SvO_2_ decrease and SvO_2_ slope was found over all measurements: the greater the deoxygenation, the steeper the drop. This is plausible by considering the similar TSSs found across trials.

### Time to task failure during different muscle actions

The TTF was the performance variable in the presented study. It appeared to be longer during PIMA compared to HIMA. However, the difference (5.51 ± 18.37 s) was not significant (methodological limitations see below). This is in line with the results of other studies which also examined the TTF of the biceps brachii muscle [11] or elbow flexor muscles [3] with similar settings for HIMA and PIMA at the same intensity (60% of the MVIC) and same forearm position (horizontal). However, during lower efforts (≤ 30% of the MVIC) of the elbow flexor muscles, the TTF of HIMA seems to be significantly shorter than the TTF of PIMA [3, 4, 9, 11]. If the isometric muscle action is, by contrast, performed in a vertical forearm position or during muscle activities at 45% and 60% of the MVIC, the TTF was found to be similar between tasks [3, 4]. This indicates that both, the intensity and forearm position, influences the performance of elbow flexor muscles. Regarding the first dorsal interosseous muscle, Maluf et al. (2005) found differences in the TTF between HIMA and PIMA at 20% of MVIC (TTF HIMA < TTF PIMA) but not at 60% [39].

However, the order of tasks in the presented study might have influenced the TTF in favor of HIMA. In case HIMA was performed at first (in 6 of 9 cases), the relation of PIMA/HIMA amounted to ~ 1.17 ± 0.68; in case PIMA was performed at first (3 of 9 cases) the relation PIMA/HIMA was ~ 1.61 ± 0.53.

Additionally, it should be mentioned again that holding tasks as performed in the present and the other mentioned studies does not imply a pure isometric muscle action. In general, muscles show slight oscillations during isometric muscle actions [2, 10, 40–44]. Thus, minor muscle shortenings and lengthenings are present. In case of weight holding, slight motions around the given joint angle have been accepted. The tolerance in different studies ranged from 2° to 10°. This also includes minor concentric contractions to lift the weight back to the starting angle position. Such muscle actions interrupt a pure HIMA and it was hypothesized that the muscle action could be switched to a PIMA, thereby [2]. In the present study, little concentric contractions were documented during four of nine HIMAs in the HP group. This also might have biased the result and might explain why the trend of a longer TTF during PIMA did not reveal statistical significance. Different experimental procedures were applied by Schaefer and Bittmann (2017, 2021) examining elbow extensors. The methods might repeal the above-mentioned problem for HIMA during weight holding [2, 10]. In the first study, a pneumatically driven measurement system was used to realize HIMA and PIMA [2]; in the second one, an interaction between two subjects comparable with arm wrestling [10]. The former study controlled for a concentric contraction (failure criterion) and the latter one facilitates the adherence to tasks (acting part: PIMA and reacting part: HIMA). The reacting (holding) subject just had to adapt to the input of the acting (pushing) partner. In both studies, the forearm was positioned vertically and significant differences regarding the TTF of elbow extensors at 80% of the MVIC (TTF HIMA < TTF PIMA) were found. Thus, not only the intensity of muscle activity and positioning but also the examined muscle and experimental procedure might play a role.

In this regard, the present study revealed that intermittent voluntary muscle twitches during a holding task extended the TTF significantly (~ 10 s). It was assumed that twitches induce a switch of the muscle action from reacting (HIMA) to acting (PIMA) during the isometric phases. Considering PIMA and Twitch together and comparing theses to all HIMA trials, the TTF still differs significantly. As discussed above, the behavior of SvO_2_ and rHb in conjunction with variables 1.–5. were similar between tasks. Thus, the longer TTF during PIMA (incl. Twitch) seems not to be derived from a different oxygen or blood supply. This is further supported by the analyzed correlations. Independently of the isometric task, very low to low, non-significant correlations between TTF and SvO_2_ decrease as well as TTF and SvO_2_ slopes (r = − 0.13 and 0.31, respectively) were found. These results indicate a reasonable independence of the TTF from the deoxygenation as long as SvO_2_ levels off into a homeostatic steady state. Booghs et al. (2012) also conclude that the decrease in muscle oxygenation is not a significant predictor of the TTF although they did not rule out its contribution to muscle fatigue [11]. Moreover, an enhancement of muscle oxygenation (oxygen half time recovery) as revealed in rock climbers during fatiguing forearm muscle contractions at 60% of the MVIC by New Zealand blackcurrant extract did not affect the TTF [45]. Consequently, there must be other factors why TTF was found to be extended during Twitch. An increased blood flow and altered muscle metabolism might play a role. In this regard, a contribution of muscle pump and vasodilation starting immediately after onset of dynamic and even single contractions has been discussed [46–49]. However, the indirectly related parameters measured in the presented study did not change subsequently to a single twitch. This could possibly be explained by the missing relaxation phase since the examined muscle had to act isometrically afterwards. Thus, we rather assume neuromuscular factors than metabolic ones.

It was previously suggested that the neuromuscular control could play a decisive role in the distinction of HIMA and PIMA [2]. This was based on the assumption that PIMA is closer to the motor control processes of a concentric contraction [2]. In contrast, HIMA might show a proximity to eccentric muscle action [2]. Eccentric muscle actions involve more complex control strategies compared to concentric ones possibly resulting in a greater central fatigue [2, 50–54]. Thus, the more complex control processes suggested for HIMA might be one reason for the often found shorter TTF compared to PIMA [2–4, 9–11]. The significantly longer TTF during Twitch in the present study, could support the assumption of a switch from HIMA to PIMA.

Because our findings should be assessed as preliminary, future investigations with larger samples are indicated to verify the results and examine other parameters in different muscles for an explanation of the potential longer TTF during PIMA. Further covariates like training status have not been considered yet and could also influence the result of TTF.

### Study limitations

Limitations regarding the used measurement technique, not examined skinfold thickness in relation to the penetration depth of the light and different determinations methods of the MVIC have been addressed previously [13, 17].

Some more limitations need to be emphasized especially regarding the TTF. During HIMA and Twitch, the loading was stopped as soon as the elbow angles exceeded 90° for more than 2 s and if twitches were not visible anymore. No objective instrument (e.g., goniometer) was used to reach highest control accuracy. The measurement error could have influenced individual trials but on group level the random error ought to be leveled out. However, the amplitude and velocity of twitches might have varied between trials. TTFs could also be influenced by the measurement position, as discussed before. Subjects used a horizontal forearm position in all measurements but changed from standing to sitting position between HIMA and PIMA. Thus, activation of trunk stabilizing and postural muscles were different between tasks with an expected higher activity during HIMA (standing). However, the biceps brachii muscle had to be equally activated with 60% of the MVIC within the muscle chain to maintain a 90° elbow flexion. If the trunk stabilizing muscles were not strong enough, the whole body would be bend forward. This was prevented by the counter bearing between the upper arm and thorax. Furthermore, due to the exclusion of measurements (see statistical analysis) the sequence of tasks of HP-group were not balanced anymore (n = 6 HIMA first; n = 3 PIMA first). Thus, effects of fatigue might have influenced the results as discussed above. In contrast, the order was still nearly balanced in HT group (n = 4 HIMA first, n = 5 Twitch first).

## Conclusions

Muscle oxygenation seem to be similar during HIMA and PIMA (especially the max. deoxygenation and oxygenation level at global minimum of blood filling of the venous capillary system). As a consequence, the behavioral pattern of the parameters SvO_2_ and rHb (type I: parallel or type II: partly inverse) occurred independently of the isometric task. In addition, intermittent voluntary muscle twitches might not alter their behavior. Possibly, oxygen and blood supply is already sufficient during isometric muscle actions without twitches. This could also explain why the TTF did not substantially correlate with the maximal deoxygenation independently of the isometric task.

In respect of the TTF, the study adds data regarding a possible objective distinction between two types of isometric contractions. The TTF tend to be shorter during HIMA compared to PIMA of the elbow flexors performed in a horizontal forearm position at 60% of the MVIC. Considering Twitch, the trend reveals statistical significance. More research is necessary in that field, especially at higher intensities (≥ 60% MVIC), different muscles and positioning.

Due to the mentioned study limitations, pilot character of the study and scarce literature on that topic, the conclusions should be seen as preliminary.

## Supplementary Information

**Additional file 1: Table S1.** Extracted data from smoothed curves (moving average, maximal smoothing width: 50).

## Data Availability

All data generated or analyzed during this study are included in this published article and supplementary material, respectively.

## Abbreviations

AU: Arbitrary units
BMI: Body mass index
EMG: Electromyography
HIMA: Holding isometric muscle action
HP: HIMA vs. PIMA group
HT: HIMA vs. Twitch group
M: Arithmetic mean
max. SvO_2_ decrease: Maximal deoxygenation
MVIC: Maximal voluntary isometric contraction
NI DIAdem^TM^: National Instruments DIAdem™
NIRS: Near infrared spectroscopy technique
O2C: Oxygen To See; LEA Medizintechnik GmbH
PIMA: Pushing or pulling isometric muscle action
rHb: Relative hemoglobin amount
RP: Reversal point
SD: Standard deviation
SvO_2_: Local capillary venous oxygen saturation of hemoglobin
SvO_2 _ at rHb min.: SvO_2_ level at global minimum of rHb
SvO_2 _ slope: Slopes of initial linear SvO_2_ decrease
TTF: Time to task failure
TSS: Time to leveling off into a steady state of SvO_2_
Twitch: Intermittent voluntary muscle twitch
