# Supplementary material for: Muscle oxygenation and time to task failure of submaximal holding and pulling isometric muscle actions and influence of intermittent voluntary muscle twitches
Source: BMC Sports Sci Med Rehabil. 2022 Mar 30;14:55. doi: 10.1186/s13102-022-00447-9 (PMC8966203; doi:10.1186/s13102-022-00447-9)
Supplement: Supplementary file 1 — Additional file 1: Table S1. Extracted data from smoothed curves (moving average, maximal smoothing width: 50). [file 13102_2022_447_MOESM1_ESM.docx]

Article

**Muscle oxygenation and time to task failure of submaximal holding and pulling isometric muscle actions and influence of intermittent voluntary muscle twitches**

(Dech S, Bittmann FN, Schaefer LV)

**Table S1.** Extracted data from smoothed curves (moving average, maximal smoothing width: 50).

| subject no._arm_task | baseline value SvO_2_ in % | max. SvO_2_ decrease  in pp | SvO_2_ slope  in pp s^–1^ | SvO_2_ at rHb min.  in % | TSS  in s | TTF  in s | MVIC in Nm | behavioral  type of SvO_2_ and rHb |
| --- | --- | --- | --- | --- | --- | --- | --- | --- |
| HP group | | | | | | | | |
| 01_ri_H | 64.01 | 13.29 | -1.00 | 58.70 | 6.93 | 45.03 | - | II |
| 01_le_H | 80.74 | 15.52 | -1.87 | 66.28 | 10.75 | 43.08 | - | I |
| 02_ri_H | 81.10 | 18.10 | -3.11 | 64.00 | 4.50 | 37.20 | - | I |
| 02_le_H | 72.86 | 14.86 | -1.77 | 62.00 | 9.70 | 43.08 | - | II |
| 03_ri_H | 78.02 | 34.02 | -5.26 | 57.00 | 4.28 | 25.45 | - | II |
| 03_le_H | 74.51 | 19.51 | -3.70 | 59.00 | 3.33 | 19.55 | - | II |
| 05_ri_H | - | - | - | - | - | - | - | - |
| 05_le_H | - | - | - | - | - | - | - | - |
| 06_ri_H | 65.78 | 11.16 | -1.19 | 60.17 | 10.45 | 74.38 | - | II |
| 06_le_H | 67.54 | 7.31 | -0.90 | 61.50 | 8.95 | 70.48 | - | I |
| 07_le_H | - | - | - | - | - | - | - | - |
| 07_le_H | 79.43 | 10.43 | -1.09 | 69.00 | 10.63 | 45.03 | - | I |
| 01_ri_P | 73.99 | 19.95 | -1.42 | 58.50 | 13.43 | 48.93 | 65.66 | II |
| 01_le_P | 82.62 | 15.56 | -1.70 | 67.23 | 11.65 | 47.94 | 63.12 | I |
| 02_ri_P | 69.81 | 9.81 | -0.80 | 62.00 | 10.35 | 58.70 | 93.38 | II |
| 02_le_P | 80.32 | 21.32 | -2.79 | 62.00 | 5.50 | 47.00 | 89.28 | II |
| 03_ri_P | 78.29 | 34.29 | -5.14 | 59.00 | 7.23 | 54.80 | 100.45 | II |
| 03_le_P | 78.08 | 38.08 | -4.85 | 58.00 | 7.60 | 48.93 | 98.98 | II |
| 05_ri_P | - | - | - | - | - | - | - | - |
| 05_le_P | - | - | - | - | - | - | - | - |
| 06_ri_P | 79.64 | 23.45 | -2.33 | 60.30 | 7.38 | 66.55 | 30.89 | II |
| 06_le_P | 73.91 | 13.70 | -1.77 | 62.76 | 6.87 | 48.12 | 33.54 | I |
| 07_ri_P | - | - | - | - | - | - | - | - |
| 07_le_P | 80.32 | 9.32 | -0.90 | 72.00 | 6.05 | 31.96 | 44.63 | I |
| **M** | **75.61** | **18.32** | **-2.31** | **62.19** | **8.09** | **47.57** | **68.88** | **-** |
| **SD** | **5.66** | **9.05** | **1.51** | **4.11** | **2.81** | **14.27** | **27.93** | **-** |
| HT group | | | | | | | | |
| 01_ri_H | 62.38 | 14.38 | -1.51 | 59.00 | 7.70 | 47.00 | 60.00 | II |
| 01_le_H | 84.07 | 24.19 | -3.26 | 60.56 | 9.70 | 48.95 | 62.50 | II |
| 02_ri_H | 78.62 | 52.11 | -6.36 | 55.93 | 5.48 | 43.08 | 72.00 | II |
| 02_le_H | 78.34 | 54.18 | -4.69 | 58.58 | 9.68 | 43.08 | 74.40 | II |
| 05_ri_H | - | - | - | - | - | - | - | - |
| 05_le_H | - | - | - | - | - | - | - | - |
| 07_ri_H | 72.05 | 30.51 | -2.71 | 55.86 | 8.28 | 35.23 | 100.80 | II |
| 07_le_H | 78.28 | 28.28 | -3.64 | 60.00 | 2.40 | 31.30 | 96.00 | II |
| 09_ri_H | 76.89 | 33.09 | -4.17 | 55.56 | 6.15 | 39.13 | 84.00 | II |
| 11_ri_H | 64.92 | 23.54 | -2.27 | 58.89 | 10.98 | 56.78 | 70.31 | II |
| 11_le_H | 75.92 | 12.67 | -1.66 | 63.58 | 8.13 | 39.15 | 66.38 | I |
| 01_ri_T | 62.38 | 7.38 | -1.49 | 59.00 | 5.18 | 48.95 | - | II |
| 01_le_T | 75.27 | 17.74 | -1.96 | 60.82 | 7.50 | 66.58 | - | II |
| 02_ri_T | 76.21 | 50.69 | -4.73 | 55.62 | 10.58 | 58.73 | - | II |
| 02_le_T | 79.85 | 53.04 | -5.11 | 55.36 | 9.70 | 37.18 | - | II |
| 07_ri_T | 74.73 | 25.44 | -3.41 | 57.64 | 5.13 | 31.33 | - | II |
| 07_le_T | 75.81 | 24.81 | -3.00 | 60.00 | 7.98 | 60.03 | - | II |
| 09_ri_T | 70.21 | 42.68 | -3.95 | 56.60 | 10.53 | 60.68 | - | II |
| 09_le_T | - | - | - | - | - | - | - | - |
| 11_ri_T | 75.17 | 41.30 | -2.71 | 59.11 | 14.43 | 54.80 | - | II |
| 11_le_T | 76.33 | 12.74 | -1.65 | 65.15 | 7.98 | 56.78 | - | I |
| **M** | **74.30** | **30.49** | **-3.24** | **58.74** | **8.20** | **47.71** | **76.27** | - |
| **SD** | **5.90** | **15.26** | **1.40** | **2.75** | **2.75** | **10.87** | **14.41** | **-** |

Abbreviations: H, holding; le, left; min., minimum; M, arithmetic mean; MVIC, maximal voluntary isometric contraction; no., number; P, pulling; pp, percent points; rHb, relative hemoglobin amount; ri, right; SD, standard deviation; SvO_2_, local capillary venous oxygen saturation; T, intermittent voluntary muscle twitch; TSS, time to leveling off into a steady state of SvO_2_; TTF, time to task failure.
